# Participation and dropout of Hockey New South Wales participants in 2017 and 2018: a longitudinal study

**DOI:** 10.1186/s13102-022-00494-2

**Published:** 2022-06-08

**Authors:** Katherine B. Owen, Bridget C. Foley, Rochelle Eime, Catriona Rose, Lindsey J. Reece

**Affiliations:** 1 SPRINTER, Prevention Research Collaboration, Charles Perkins Centre, School of Public Health, Faculty of Medicine and Health, The University of Sydney, Level 6, The Charles Perkins Centre, Camperdown, NSW 2006 Australia; 2 Institute of Health and Wellbeing, Federation University, Ballarat, VIC Australia; 3 Institute for Health and Sport, Victoria University, Melbourne, VIC Australia

**Keywords:** Sport, Physical activity, Drop out, Adolescents, Children, Adults

## Abstract

**Background:**

Sports have a focus on increasing participation, which contributes to increasing population levels of physical activity, social cohesion and longevity of the sport. The primary aim of this study was to examine reasons for drop-out of a popular team sport in Australia, Field Hockey and identify opportunities to increase participation.

**Methods:**

This longitudinal study obtained routinely collected registered player data from Hockey New South Wales over two consecutive years, and survey data from registered players who dropped out. Logistic regression models identified demographic subgroups who were more likely to drop out of sport, and the reasons for dropping out.

**Results:**

In 2018, 8463 (31%) of hockey players did not return to play hockey after the previous season and 805 (10%) of these completed a survey. Specific groups who were more likely to stop playing included 5–6 years (OR: 2.1, 95% CI 1.8–2.6; reference: 12–17 years), females (OR: 1.1, 95% CI 1.0–1.2; reference: males), Indigenous (OR: 1.2, 95% CI 1.1–1.4; reference: non-Indigenous), most disadvantaged (OR: 1.1, 95% CI 1.0–1.2; reference: least disadvantaged) or regional and remote (1.1, 95% CI 1.0–1.2; reference: major cities). Top reasons for drop out were medical/age (17%), change in circumstances (16%) and high cost (13%), lack of time (13%) and lack of enjoyment (7%).

**Conclusions:**

Although Hockey successfully reaches a large proportion of underrepresented groups in sport, these groups are more likely to drop out. Sports should consult these groups to develop enjoyable, flexible, and modifiable versions of the game that are appropriate to their needs.

## Background

Regular participation in physical activity provides various physical and mental health benefits for all age groups [1, 2]. The benefits of team sport extend further than those of individual types of physical activity, as a result of the additional psychological and social benefits of being part of a team or sports club [2, 3]. For children and adolescents, the social nature of team sport may improve self-esteem, social skills, confidence, competence and reduces depressive symptoms [2]. For adults, sport participation within a team can provide emotional social support, sense of belonging, higher self-esteem, social network and reduce stress and anxiety [4]. On a community level, sport can contribute by bringing the community together and increasing social cohesion in crisis situations (e.g. Coronavirus **[**COVID-19]) [5]. In Australia, it is estimated that sport creates over $29 billion of benefits through reduced healthcare costs and reduced early mortality annually [6]. It is therefore critical for economic, social and health benefits to increase sport participation.

The World Health Organisation has recognised that sport is a central element of increasing population levels of physical activity [7]. Increasing physical activity within each sport is a specific focus of sports governing organisations. Studies examining sport participation have found that participation peaks at around 10–11 years [8–10], and then declines throughout adolescence [11] and adulthood [8, 10]. This peak can partially be explained by children tending to sample multiple sports, and then at approximately 13 years, choosing one sport to specialise in, or dropping out altogether [11]. The Developmental Model of Sport Participation suggests that is beneficial for 6–12 year olds to sample multiple sports as it can reinforce a range of physical, personal, and mental skills required for future sport success [12]. In contrast, early specialisation in one sport often leads to dropout due to the heavy training, burnout, injures and a decrease in performance from to overtraining [13–15]. Other common reasons associated with drop out among children are a lack of physical or sport competence and limited enjoyment of sport [16]. Children who have higher perceived competence or actual competence are more likely to enjoy the sport and be intrinsically motivated. Intrinsic motivation exists when the behaviour is viewed as interesting or enjoyable and leads to long-term participation [17]. The development and implementation of strategic actions which address these reasons are required to reduce sport drop out.

Designing effective national and sub-national sports policy and participation strategies continues to be a challenge in Australia due to a historical lack of sport participation data that is representative of all Australian’s participating in sport. With some exceptions [e.g., 8], most studies are limited to small self-report samples that are subject to response bias [18]. Sport specific registration data is effectively a census of all members and can provide valuable information about participation trends. Furthermore, sport-specific registration data is currently underutilised and, if used critically, could inform national sport policy and participation strategies [8].

Field hockey is a popular sport worldwide and in Australia, with approximately 65,000 Australians playing each year and one quarter of these playing in New South Wales (NSW) [10]. Field hockey is a high intensity team sport played by people of all ages and levels, ranging from amateur to the elite level [19]. The sport involves high-speed running with accelerations, decelerations, changes in direction, and tactical skills, with the aim to outscore the opposition [20]. Field hockey can be played on multiple surfaces, some specialised for the sport (watered turf, artificial/synthetic field), and others multi-purpose (grass, indoor boarded surface). To play field hockey, equipment is required including a hockey stick and protective gear (e.g., mouth guard and shin pads). There is a paucity of research in recreational or community-level hockey participants [e.g., 21], with the focus of studies largely being on elite athlete [22]. As with many team sports, the population who play hockey are predominately community-level players and it is therefore important to understand how to retain or increase the number of participants at this level. The aim of this study was to examine participation and drop-out of field hockey in NSW using longitudinal data that is representative of hockey participants. Secondly, this study explored the reasons for drop-out to inform strategies that Hockey NSW and other sporting organisations could use to reduce drop-out.

## Methods

This longitudinal study obtained routinely collected participant registration data from Hockey NSW over two consecutive years, and a cross-sectional survey. The University of Sydney Human Research Ethics Committee granted ethics approval for this study (Protocol number: 2020/732).

A field hockey participant was defined as a participant registered with Hockey NSW, the peak body for Field Hockey in NSW. Hockey NSW provided routinely collected participant registration data for all hockey participants in 2017 and 2018. Routinely collected registration data included their unique sport membership ID, and the compulsory questions asked at registration including date of birth, sex, parent’s country of birth, disability status (Yes/No/Do not wish to disclose), Indigenous status (Yes/No/ Do not wish to disclose), and residential postcode. This data is a census of most field hockey participants in NSW and includes all ages (i.e., juniors, adults, masters and seniors) and all versions of the game (i.e., indoor and outdoor school competitions, and modified versions).

Postcode of residence was used to determine socioeconomic status (SES) using the Socio-Economic Index for Area (SEIFA), specifically the Index of Relative Socio-Economic Disadvantage [23]. SEIFA ranks regions in Australia based on relative socioeconomic disadvantage into percentiles, which were converted into quartiles ranging from 1 (most disadvantaged) to 4 (least disadvantaged). Postcode was also used to categorise location (i.e., metropolitan vs regional and remote areas) using the Accessibility and Remoteness Index of Australia [24].

Players who participated in 2017 and did not register and participate in 2018, were categorised as having dropped out of field hockey participation through linking the Hockey NSW 2017 and 2018 data. These members (n = 8463) were invited to participate in an online survey by Hockey NSW during September 2018. The survey was designed to be completed independently by hockey registrants over the age of 16 years, or by children with the support of their parent/carer. The first question asked: “What is the main reason for no longer playing hockey?” with response options: (a) change in circumstances, (b) High cost/low value, (c) Loss of interest in hockey, (d) Medical/age, (e) No time/time constrains, (f) Poor experience, (g) Scheduling, (h) no 'social' version of the game offered, (i) Travel, or (j) Other. The second questions asked: “We really miss you, what is the one thing hockey could do to get you back?” with options: (a) Reduce cost, (b) Improve value for money, (c) Provide discounts for multiple family members, (d) Provide opportunity to become an umpire or official, (e) Provide opportunity to volunteer at a club/association, (f) Provide different playing options that are more flexible, (g) Improve professionalism and management of association/club, (h) Make the association/club more welcoming and tolerant, (i) Provide training and playing fields closer to where I live, or (j) Other. Participation was voluntary and members who participated were entered in a draw to win one of 2x $100 Sport gift cards.

### Data analysis

Descriptive statistics, including frequencies and proportions were calculated for participants in 2017 and 2018. The 2017 and 2018 registration data was linked using participant’s unique player registration ID. We conducted a series of binary logistic regression models to determine the odds ratio (OR) and 95% confidence interval (CI) for dropping out of hockey for each demographic subgroup. We set the reference group to be the subgroup with higher levels of sport participation (e.g., 12–17 years and males) [8]. All analyses were performed in SAS Enterprise Guide 9.4 (SAS Institute, Cary, NC, USA).

## Results

### Demographic characteristics of Hockey NSW participants in 2018

The total number of participants was similar across 2017 (N = 27,534) and 2018 (N = 26,826) (Table 1). Of these, almost half were aged 0–17 years (2017: 47%; 2018: 46%) and more than half were females (2017 and 2018: 58%). Some participants identified as Indigenous (2017: 5%; 2018: 4%) or having a disability (2017 and 2018: 1%). A greater proportion of participants lived in least disadvantaged areas (2017: 24%; 2018: 25%) compared with the most disadvantaged areas (2017: 15%; 2018: 16%).

**Table 1:** Hockey NSW registration demographics and the odds of dropping out in the 2018 season

	2017 registrations		2018 registrations		Odds of dropping out in 2018
	N	%	N	%	OR (95% CI)
All registrants	27,534	100	26,826	100	
Age category					
5–6 years	1091	4.0	914	3.4	2.1 (1.8, 2.4)
7–11 years	5289	19.2	5076	18.9	0.8 (0.8, 0.9)
12–17 years	6547	23.8	6369	23.7	Reference
18–25 years	3944	14.3	3812	14.2	1.6 (1.4, 1.7)
25–34 years	3253	11.8	3143	11.7	1.3 (1.2, 1.4)
35–44 years	3251	11.8	3185	11.9	0.9 (0.8, 1.0)
45–54 years	2739	10.0	2802	10.5	0.8 (0.7, 0.8)
55–64 years	1112	4.0	1187	4.4	0.6 (0.5, 0.7)
65 + years	308	1.1	337	1.3	0.9 (0.7, 1.1)
Sex					
Male	11,319	41.1	11,200	41.8	Reference
Female	16,057	58.3	15,535	57.9	1.1 (1.0, 1.2)
Missing	158	0.6	91	0.3	
Country of birth					
Australia	15,213	55.3	21,367	79.7	Reference
Other	2577	9.4	3864	14.4	1.3 (0.6, 2.8)
Missing	9744	35.4	1595	6.0	
A parent born overseas					
Yes	3189	11.6	4855	18.1	1.1 (0.5, 2.2)
No	11,938	43.4	17,075	63.7	Reference
Missing	12,407	45.1	4896	18.3	
Identified as Indigenous					
Yes	1231	4.5	1180	4.4	1.2 (1.1, 1.4)
No	25,950	94.3	25,333	94.4	Reference
Missing	353	1.3	313	1.2	
Identified as having a Disability					
Yes	305	1.1	337	1.3	0.7 (0.5, 0.9)
No	26,910	97.7	26,239	97.8	Reference
Missing	319	1.2	250	0.9	
Socio-economic status					
1st quartile (most disadvantaged)	4244	15.4	4221	15.7	1.1 (1.0, 1.2)
2nd quartile	9429	34.2	9130	34.0	1.1 (1.0, 1.2)
3rd quartile	6682	24.3	6550	24.4	1.1 (1.0, 1.1)
4th quartile (least disadvantaged)	6661	24.2	6569	24.5	Reference
Missing	518	1.9	356	1.3	
Location					
Major cities	12,612	45.8	12,358	46.1	Reference
Inner regional	12,356	44.9	12,048	44.9	1.1 (1.1, 1.2)
Outer regional and remote	2124	7.7	2137	8.0	1.1 (1.0, 1.2)
Missing	442	1.6	283	1.1	

OR: Odds ratio; CI: confidence interval. This table uses registration data only

### Demographic characteristics of participants who dropped out of Hockey in 2018

In 2018, 8463 (31%) of Hockey NSW players did not return to play hockey after the 2017 season (Table 2). Compared with the 12–17 year age group, the 5–6 year age group were twice as likely to drop out (OR: 2.1, 95% CI 1.8–2.4) and the 18–34 year age groups were more likely to drop out of hockey (18–25 years OR: 1.6, 95% CI 1.4–1.7; 26–34 years OR: 1.3, 95% CI 1.2–1.4). Females were 9% more likely to drop out compared with males (OR: 1.1, 95% CI 1.0–1.2). Participants who identified as Indigenous were 21% more likely to drop out compared to those who did not (OR: 1.2, 95% CI 1.1–1.4). Participants with a disability were 32% less likely to drop out compared to those without a disability (OR: 0.7, 95% CI 0.5–0.8). Those in disadvantaged area and regional and remote areas were approximately 10% more likely to drop out (OR: 1.1, 95% CI 1.0–1.2 and OR: 1.1, 95% CI 1.0–1.2, respectively).

**Table 2:** Hockey NSW drop out registration and survey demographics

	2018 registration dropouts		2018 registration dropouts who completed a survey		Response rate
	N	%	N	%	%
All registrants	8463	100.0	805	100.0	9.5
Age category					
0–17 years	3862	45.6	258	32.1	6.7
18–25 years	1574	18.6	140	17.4	8.9
26–34 years	1152	13.6	88	10.9	7.6
35–44 years	889	10.5	128	15.9	14.4
45–54 years	673	8.0	117	14.5	17.4
55–64 years	231	2.7	51	6.3	22.1
65 + years	82	1.0	23	2.9	28.0
Sex					
Male	3321	39.9	330	41.0	9.9
Female	4993	60.1	475	59.0	9.5
Socio-economic status					
1st quartile (most disadvantaged)	1316	15.6	111	13.8	8.4
2nd quartile	2888	34.1	281	34.9	9.7
3rd quartile	2001	23.6	204	25.3	10.2
4th quartile (least disadvantaged)	1933	22.8	203	25.2	10.5
Location					
Major cities	3657	44.8	384	47.7	10.5
Inner regional	3847	47.1	357	44.4	9.3
Outer regional and remote	667	8.2	62	7.7	9.3

Age was categorised in a multiple-choice question in the survey and therefore cannot be broken down any further

### Survey responses

Of the 8463 hockey participants who dropped out after the 2017 season, 805 completed a survey (response rate: 9.5%). The response rate increased with age, with the highest rate in the 65 + year age group (28%). Those living in the least disadvantaged areas were also more likely to respond to the survey (11%) compared to those living in the most disadvantaged areas (8%).

### Reasons for drop out

The most common reasons for dropping out of hockey were medical/age (17%), change in circumstances (16%), high cost/low value (13%), no time (12%) and a loss of interest (7%) (Table 3).

**Table 3:** Odds of reporting the top reasons for drop out by demographic characteristics

	Medical/age (N = 133; 17%) OR (95% CI)	Change in circumstances (N = 126; 16%) OR (95% CI)	High cost (N = 105; 13%) OR (95% CI)	No time N = 99; 12%) OR (95% CI)	Loss of interest (N = 57; 7%) OR (95% CI)
Age category					
0–17 years	Reference	Reference	Reference	Reference	Reference
18–25 years	1.8 (0.8, 4.1)	4.3 (2.4, 7.6)	3.3 (1.8, 6.3)	1.2 (0.7, 2.2)	0.2 (0.1, 0.5)
26–34 years	2.1 (0.8, 5.2)	4.5 (2.4, 8.5)	3.7 (1.8, 7.4)	1.3 (0.7, 2.7)	0.1 (0.0, 0.4)
35–44 years	5.0 (2.4, 10.3)	2.5 (1.3, 4.6)	2.3 (1.2, 4.6)	1.1 (0.6, 2.1)	0.1 (0.0, 0.4)
45–54 years	11.5 (5.8, 22.9)	0.8 (0.3, 1.8)	1.8 (0.9, 3.8)	1.4 (0.7, 2.6)	0.2 (0.1, 0.5)
55 + years	18.4 (8.8, 38.5)	1.0 (0.4, 2.4)	1.4 (0.6, 3.5)	0.3 (0.1, 1.1)	0.1 (0.0, 0.5)
Sex					
Male	Reference	Reference	Reference	Reference	Reference
Female	0.8 (0.6, 1.2)	2.0 (1.3, 3.0)	0.9 (0.6, 1.4)	1.1 (0.7, 1.7)	1.0 (0.6, 1.6)
Socioeconomic status					
1st quartile (most disadvantaged)	1.4 (0.8, 2.6)	0.7 (0.3, 1.4)	1.6 (0.8, 3.1)	0.5 (0.2, 1.0)	2.4 (1.0, 5.9)
2nd quartile	1.3 (0.8, 2.1)	1.3 (0.8, 2.2)	1.4 (0.8, 2.5)	0.7 (0.4, 1.2)	1.2 (0.5, 2.8)
3rd quartile	1.2 (0.7, 2.0)	1.2 (0.7, 2.0)	1.2 (0.7, 2.2)	0.8 (0.4, 1.3)	2.6 (1.2, 5.8)
4th quartile (least disadvantaged)	Reference	Reference	Reference	Reference	Reference
Location					
Major cities	Reference	Reference	Reference	Reference	Reference
Inner regional	1.10 (0.7, 1.6)	0.8 (0.5, 1.2)	1.4 (0.9, 2.2)	0.6 (0.4, 0.9)	2.1 (1.2, 3.9)
Outer regional and remote	0.9 (0.4, 1.9)	2.8 (1.6, 5.1)	1.0 (0.4, 2.4)	0.48 (0.2, 1.3)	1.8 (0.6, 5.0)

OR: Odds ratio; CI: confidence interval. Age was categorised in a multiple-choice question in the survey and therefore cannot be broken down any further. The 55–64 and 65 + year age groups were combined due to small numbers

Of those who indicated medical/age explained why they had not returned, 75% elaborated in the free text box and reported an injury, 15% specified an illness and 10% stated increasing age. The odds of reporting medical/age significantly increased with age, with the 55 + year age group being 18 times more likely to drop out for medical/age reasons compared with the 0–17-year age group (OR: 18.4, 95% CI 8.8–38.5).

Change in circumstances included moving interstate (61%) or overseas (16%), and pregnancy (23%). Change in circumstance was a more common reason for dropout among 18–34 years compared with 0–17 years (18–25 years OR: 4.3, 95% CI 2.4–7.6; 26–34 years OR: 4.5, 95% CI 2.4–8.5), for females compared with males (OR: 2.0, 95% CI 1.3–3.0), and for outer regional and remote areas compared with major cities (OR: 2.8, 95% CI 1.6–5.1).

Participants expanded on high cost and indicated that the cost of participation was too high (81%) and it put a strain on the family budget (19%). High cost was more likely to be reported by 18–44 years compared with 0–17 years (18–25 years: OR 3.3, 95% CI 1.8–6.3; 26–34 years OR: 3.7, 95% CI 1.8–7.4; 35–44 years OR: 2.3, 95% CI 1.2–4.6).

Those who reported they ‘lost interest in hockey’ (7%) had other recreational commitments (40%), or other sporting commitments (60%). The 0–17-year age group was significantly more likely to report losing interest in hockey compared to all other age groups. The most disadvantaged group were also more likely to report losing interest compared to the least disadvantaged group (OR: 2.4, 95% CI 1.0–5.9) and participants living in inner regional areas were more likely to report losing interest compared with participants living in major cities (OR: 2.1, 95% CI 1.2–3.9).

### Strategies to prevent drop out

Hockey participants who dropped out of the sport suggested that Hockey NSW might get them to return to play if they reduced the cost (31%), did nothing at this point in time (15%) or provided additional hockey fields for training and games closer to where the registrant lives (9%). Suggesting that hockey should lower the costs was reported by a higher proportion of registrants living in the most disadvantages areas (35%) compared to those living in the least disadvantaged areas (26%). Half (51%) of the 18–25-year age group reported that lowering the cost of hockey would get them to return to play compared with 16% of people in the 0–17 year age group.

## Discussion

This study utilized registration data over a two-year period from the main sporting organization for field hockey in NSW, to examine sport participation and dropout. In 2018, one third (31%) of hockey registrants did not return after the 2017 season. We found that groups who were most likely to drop out included the 5–6 year and 18–35 year age groups, females, Indigenous, disadvantaged and regional or remote areas. Top reasons for drop out were medical/age, change in circumstances, high cost, and lack of time. Understanding key demographic groups that do not return to the sport, as well as motives for dropping out, can unlock the potential for new strategic plans and policies to increase participation in hockey, and other sports.

Hockey NSW reaches a large proportion of participants who are underrepresented in sports participation, including females, Indigenous, disadvantaged, and regional and remote areas. A positive finding of this study was that females made up 58% of all hockey participants in 2017 and 2018 and were significantly less likely to drop out of hockey compared with men. Reducing the prevalence of inactivity in women, without changing the prevalence in men, would achieve the 2025 WHO global target for inactivity [25]. This is a positive finding and hockey should continue to strive for gender equity within the sport.

Although Hockey NSW successfully reaches a large proportion of underrepresented groups in sport, these groups are more likely to drop out (with the exception of females). To effectively retain these population groups in sport, Hockey NSW (and all sports) should adapt elements of their sport delivery. Staley et al. (2019) suggests sports should recruit appropriate product deliverers, build the capacity of the delivery organisation, and develop relevant social sport products which align with the specific needs and characteristics of the target groups [26]. For example, Peralta, Cinelli (28) conducted a sport-based mentoring program with remote Aboriginal communities in Australia. All parts of the program design and evaluation were conducted in consultation with an Aboriginal organisation and Aboriginal community members within each community to ensure that the program met each specific school and student needs. This resulted in the program successfully engaging Aboriginal youth living in remote communities. National and state sporting organisations should consult with community groups that advocate for these underrepresented sections of society to develop sport products that are popular among the cohort and appropriate to their needs.

People living with a disability are underrepresented in Hockey, but when they do register, they are less likely to drop out compared with registrants without a disability. Only 1.3% of hockey registrants identified as having a disability, whereas 18% of the Australian population are estimated to have a disability [28]. People living with disabilities face additional barriers to participation in team sports, compared to those without a disability such as a lack of disability-trained coaches/instructors, unwillingness to be inclusive, negative societal attitudes towards disability, and a lack of local opportunities [29]. To increase representativeness of people living with disabilities, multiple collaborative approaches are needed. For example, disability organisations could collaborate with state and national sports organisations to develop introductory programs for children with disabilities that could be delivered in schools, increasing the accessibility of sport to this cohort. Respondants living with a disability who registered in 2017 were more likely to reregister in 2018. Data from these participants provides a unique rich source of data on retention in sport for people living with disabilities. Future studies should conduct qualitative studies on why people living with disabilities become engaged and remain in sport.

Young children (5–6 years) were significantly more likely to drop out of hockey compared to any other age group. The top reason for dropout among this age group was losing interest because they had other sporting commitments, which is consistent with a recent systematic review [16]. These findings could be partially explained by children of this age sampling multiple different sports before deciding which one they want to specialize and focus on [11]. Ensuring that children enjoy sport and promoting the sampling of different sports could lead to increased long-term sport participation. Adults aged 18–35 were more likely to drop out of hockey than adolescents, where the highest rates of drop out are usually observed.

Cost was a common reason for drop out, particularly for people living in the most disadvantaged areas. This is consistent with the findings of a recent systematic review that identified cost as one of the top barriers to sport [30]. The cost of participating in some sports can be high and often includes registration, uniforms and sporting equipment. Overcoming the cost barrier, while remaining financially viable is a challenge for sports to overcome. Governments can assist sports organisations through the implementing voucher programs and tax rebates which aim to reduce the cost of sports participation. Although promotion of such initiatives to individuals living in disadvantaged areas should be strengthened [31, 32]. Sport voucher programs are only one part of a multi-component, approach to improving accessibility to sport participation, however they may allow organsiations to focus on overcoming remaining barriers. National sporting organisations, such as Sport Australia should work with the sector to develop partnerships across government and industries such as health, education, retail, digital and science to create new opportunities for investment in sport.

The strengths of this study include using the registration data over a two-year period from the peak body for field hockey in NSW. Drawing from a sample of approximately 65,000 hockey participants each year, this data set represents most hockey participants in the state. This data is a key asset to interpretation of these findings as it provides an accurate representation of players, and a stronger evidence base for decision making for the sport of field hockey. A limitation of this study was that we could not determine whether hockey members who dropped out started participating in another sport or stopped sport participation altogether. It is typical for young children to drop out and trial other sports; therefore, would be beneficial to collect more than two-years of data and data from different sports to identify different participation trajectories. Future studies should obtain longitudinal data over at least 3 years. The short surveys provide in depth understanding of why participants dropped out and make clear recommendations on how Hockey NSW could try to increase participation. We recommend that surveys like this one are sent to all hockey members on a longitudinal basis to inform future strategic decisions. Future surveys should use standardised questions to enhance the comparability of the survey data to other states in Australia or other sports. A limitation of this study was the broad age ranges collected within the survey. Future surveys should collect more detailed age data to allow comparisons with the registration data and enable a comprehensive understanding of how drop out changes across the lifespan. Another limitation of this study was the low response rate, especially in the younger age groups and most disadvantaged areas. Therefore, caution is warranted when interpreting the survey results and future studies should implement strategies to increase response rates amongst the younger age groups and most disadvantaged areas to provide a more representative survey sample.

It is timely to note the impact that COVID-19 has had on community sport, with social and physical distancing measures, lockdowns of small businesses, schools and overall social activities, and disruptions to many regular aspects of life, including sport and physical activity. As these measures and lockdowns begin to lift, understanding how to engage people in sport, maintain their engagement, and preventing drop-out is essential, not just for participant outcomes but sport sustainability.

## Conclusions

Increasing and maintaining sport participation is a priority for sports. Although Hockey successfully reaches a large proportion of underrepresented groups in sport (children and young adults, females, Indigenous, disadvantaged and regional or remote areas), these groups are more likely to drop out. Sports should consult these groups to develop enjoyable, flexible, and modifiable versions of the game that are appropriate to their needs.

## Data Availability

The datasets used and/or analysed during the current study are available from the corresponding author on reasonable request.

## References

[1] Biddle SJ, Ciaccioni S, Thomas G, Vergeer I (2019). Physical activity and mental health in children and adolescents: an updated review of reviews and an analysis of causality. Psychol Sport Exerc.

[2] Eime R, Young J, Harvey J, Charity M, Payne W (2013). A systematic review of the psychological and social benefits of participation in sport for children and adolescents: informing development of a conceptual model of health through sport. Int J Behav Nutr Phys Act.

[3] Zuckerman SL, Tang AR, Richard KE, Grisham CJ, Kuhn AW, Bonfield CM (2021). The behavioral, psychological, and social impacts of team sports: a systematic review and meta-analysis. Phys Sportsmed.

[4] Andersen MH, Ottesen L, Thing LF (2019). The social and psychological health outcomes of team sport participation in adults: an integrative review of research. Scand J Public Health.

[5] Women’s Refugee Commission U, and, GRYC. “We believe in youth”: global refugee youth consultations final report https://www.womensrefugeecommission.org/youth/resources/1385-gryc-final-report-sept-2016. 2016.

[6] Australian Sports Commission. Intergenerational review of Australian sport. Canberra, Australia, The Boston Consulting Group2017.

[7] World Health Organization (2019). Global action plan on physical activity 2018–2030: more active people for a healthier world.

[8] Eime R, Harvey J, Charity M, Casey M, Westerbeek H, Payne W (2016). Age profiles of sport participants. BMC Sports Sci Med Rehabil.

[9] Vella SA, Schranz NK, Davern M, Hardy LL, Hills AP, Morgan PJ (2016). The contribution of organised sports to physical activity in Australia: Results and directions from the Active Healthy Kids Australia 2014 Report Card on physical activity for children and young people. J Sci Med Sport.

[10] Sport Australia. AusPlay survey results July 2019 - June 2020. In: Australian Sports Commission, editor. Canberra: Australian Sports Commission,; 2020.

[11] Eime R, Harvey J, Charity M (2019). Sport drop-out during adolescence: is it real, or an artefact of sampling behaviour?. Int J Sport Policy Politics.

[12] Côté J, Vierimaa M (2014). The developmental model of sport participation: 15 years after its first conceptualization. Sci Sports.

[13] Summit ACRNYESS, Tenforde AS, Montalvo AM, Nelson VR, Myer GD, Brenner JS, et al. Current sport organization guidelines from the AMSSM 2019 youth early sport specialization research summit. Sports Health. 2022;14(1):135–41. 10.1177/19417381211051383 PMC8669930 34668454

[14] Cornelissen M, Kemler E, Verhagen E, Gouttebarge V (2020). A systematic review of injuries in recreational field hockey: From injury problem to prevention. J Sports Sci.

[15] Myer GD, Jayanthi N, Difiori JP, Faigenbaum AD, Kiefer AW, Logerstedt D (2015). Sport specialization, part I: does early sports specialization increase negative outcomes and reduce the opportunity for success in young athletes?. Sports health.

[16] Crane J, Temple V (2015). A systematic review of dropout from organized sport among children and youth. Eur Phys Educ Rev.

[17] Deci E, Ryan R (1985). Intrinsic motivation and self-determination in human behavior.

[18] Brooks MA, Post EG, Trigsted SM, Schaefer DA, Wichman DM, Watson AM (2018). Knowledge, attitudes, and beliefs of youth club athletes toward sport specialization and sport participation. Orthop J Sports Med.

[19] Jennings DH, Cormack SJ, Coutts AJ, Aughey RJ (2012). International field hockey players perform more high-speed running than national-level counterparts. J Strength Condit Res.

[20] Forouhandeh A. Establishing running intensities of elite field hockey players during competitive match-play. Int J Strength Condition. 2020;1(1).

[21] Brooke HL, Corder K, Griffin SJ, van Sluijs EM (2014). Physical activity maintenance in the transition to adolescence: a longitudinal study of the roles of sport and lifestyle activities in British youth. PLoS ONE.

[22] Delfino Barboza S, Nauta J, van der Pols M, van Mechelen W, Verhagen E (2018). Injuries in Dutch elite field hockey players: a prospective cohort study. Scand J Med Sci Sports.

[23] Australian Bureau of Statistics. Census of population and housing: socio-economic indexes for areas (SEIFA) - Technical paper, 2006 (No.2033.0.55.001). Canberra: Australian Bureau of Statistics; 2016.

[24] Australian Bureau of Statistics. Australian Statistical Geography Standard (ASGS): Volume 5 - Remoteness Structure, July 2016. (Canberra. 1270.0.55.005)2018.

[25] Mielke GI, da Silva ICM, Kolbe-Alexander TL, Brown WJ (2018). Shifting the physical inactivity curve worldwide by closing the gender gap. Sports Med.

[26] Staley K, Donaldson A, Randle E, Nicholson M, O’Halloran P, Nelson R (2019). Challenges for sport organisations developing and delivering non-traditional social sport products for insufficiently active populations. Aust N Z J Public Health.

[27] Peralta L, Cinelli R, Bennie A (2018). Mentoring as a tool to engage Aboriginal youth in remote Australian communities: a qualitative investigation of community members, mentees, teachers, and mentors’ perspectives. Mentor Tutor Partnersh Learn.

[28] Australian Bureau of Statistics. Disability, Ageing and Carers, Australia: Summary of Findings. Retrieved on 1 December 2020 from https://www.abs.gov.au/statistics/health/disability/disability-ageing-and-carers-australia-summary-findings/2018; 2018.

[29] Shields N, Synnot A (2016). Perceived barriers and facilitators to participation in physical activity for children with disability: a qualitative study. BMC Pediatr.

[30] Somerset S, Hoare DJ (2018). Barriers to voluntary participation in sport for children: a systematic review. BMC Pediatr.

[31] Owen KB, Foley BC, Bauman A, Bellew B, Reece LJ (2020). Parental awareness and engagement in the Active Kids program across socioeconomic groups. J Sci Med Sport.

[32] Foley BC, Owen KB, Bellew W, Wolfenden L, Reilly K, Bauman AE (2020). Physical activity behaviors of children who register for the universal, state-wide active kids voucher: who did the voucher program reach?. Int J Environ Res Public Health.

